# Feasibility of Piezoelectric Endoscopic Transsphenoidal Craniotomy: A Cadaveric Study

**DOI:** 10.1155/2014/341876

**Published:** 2014-02-09

**Authors:** Peter Valentin Tomazic, Verena Gellner, Wolfgang Koele, Georg Philipp Hammer, Eva Maria Braun, Claus Gerstenberger, Georg Clarici, Etienne Holl, Hannes Braun, Heinz Stammberger, Michael Mokry

**Affiliations:** ^1^Department of General Otorhinolaryngology, Head and Neck Surgery, Medical University of Graz, Auenbruggerplatz 26/28, 8036 Graz, Austria; ^2^Rhinoneurosurgery Group, Medical University of Graz, 8036 Graz, Austria; ^3^Department for Neurosurgery, Medical University of Graz, Auenbruggerplatz 29, 8036 Graz, Austria

## Abstract

*Objective*. Endoscopic transsphenoidal approach has become the gold standard for surgical treatment of treating pituitary adenomas or other lesions in that area. Opening of bony skull base has been performed with burrs, chisels, and hammers or standard instruments like punches and circular top knives. The creation of primary bone flaps—as in external craniotomies—is difficult.The piezoelectric osteotomes used in the present study allows creating a bone flap for endoscopic transnasal approaches in certain areas. The aim of this study was to prove the feasibility of piezoelectric endoscopic transnasal craniotomies. *Study Design*. Cadaveric study. *Methods*. On cadaveric specimens (*N* = 5), a piezoelectric system with specially designed hardware for endonasal application was applied and endoscopic transsphenoidal craniotomies at the sellar floor, tuberculum sellae, and planum sphenoidale were performed up to a size of 3–5 cm^2^. *Results*. Bone flaps could be created without fracturing with the piezoosteotome and could be reimplanted. Endoscopic handling was unproblematic and time required was not exceeding standard procedures. *Conclusion*. In a cadaveric model, the piezoelectric endoscopic transsphenoidal craniotomy (PETC) is technically feasible. This technique allows the surgeon to create a bone flap in endoscopic transnasal approaches similar to existing standard transcranial craniotomies. Future trials will focus on skull base reconstruction using this bone flap.

## 1. Introduction

Over the last two decades endoscopic transsphenoidal skull base approaches have become the gold standard in the surgical treatment of selected cases of pituitary adenomas and many other lesions in that area like meningiomas, chordomas, or craniopharyngiomas. However, there are still some indications for other surgical routes (transcranial, combined, etc.) in the treatment of such lesions. Extended approaches to the suprasellar and parasellar regions, planum sphenoidale, and the clivus were developed and the cooperation between ENT surgeons and Neurosurgeons was intensified [1–4]. After opening the sphenoid sinus through both natural ostia the bone of the skull base can be removed to expose and open the dura if required. The extent of bone removal is tailored to the size and localization of the lesion and can be modified for transtubercular, transplanum, and transcribiform approaches. For thick bone, burrs are used whereas thinner bone can be excised by classical punches. In our department the defect in skull base is reconstructed with hemostyptic material only or with fascia lata or even nasoseptal flaps in case of intraoperative CSF leak [5, 6]. Reconstruction as in transcranial craniotomies with osseous flaps cannot be achieved due to the preceding resection of the bone.

Especially in endoscopic pituitary surgery a craniotomy harvesting a bone flap which can later be used for skull base reconstruction could be desirable to facilitate defect closure. Until now this was rarely possible, because with standard instruments the bone would either be excised, fractured, or turned to bone dust by burrs and drills.

The advent of piezosurgical techniques could also be implied in endoscopic transsphenoidal skull base surgery. The piezoelectric effect was first described by Jacques and Pierre Curie in 1880 implying that under a mechanical force certain crystalline minerals become electrically polarized. Gabriel Lippmann later described the converse piezoelectric effect: when one of these crystalline minerals is exposed to an electric field (alternating current, AC) it is lengthened and shortened according to the polarity of the field in proportion to the field strength (Volt/meter) and frequency (Hertz, Hz) [7]. High frequency oscillations of the piezoelectric crystals are used to “cut” bone that way like microsaws [8]. The advantage of this technique is that the cutting effect is reserved to hard, crystalline mineral that is structured like bone, soft tissue like dura would not be affected because of its elasticity, as long as no additional pressure is applied [9, 10].

Initially, Piezosurgery was mainly used by maxillofacial surgeons, but nowadays it is more frequently applied for craniotomies [9–11]. Nordera et al. used it for external orbital decompression in Basedow's disease [9].

As of today piezosurgery was never used in endoscopic transsphenoidal approaches to the pituitary or anterior skull base. To our knowledge, this is the first study on the feasibility of piezoelectric endoscopic transsphenoidal craniotomies (PETC) in cadaveric specimens.

## 2. Material and Methods

**Figure 1:**
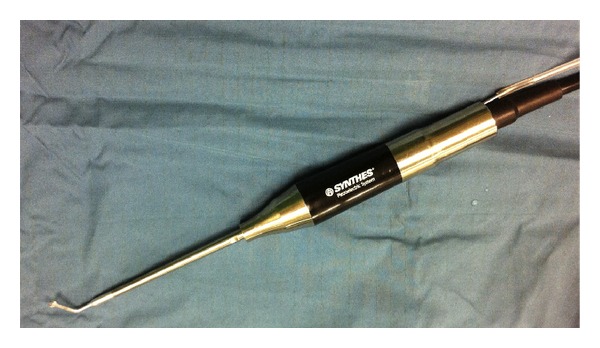
Piezoelectric handpiece suitable for endoscopic application.

**Figure 2:**
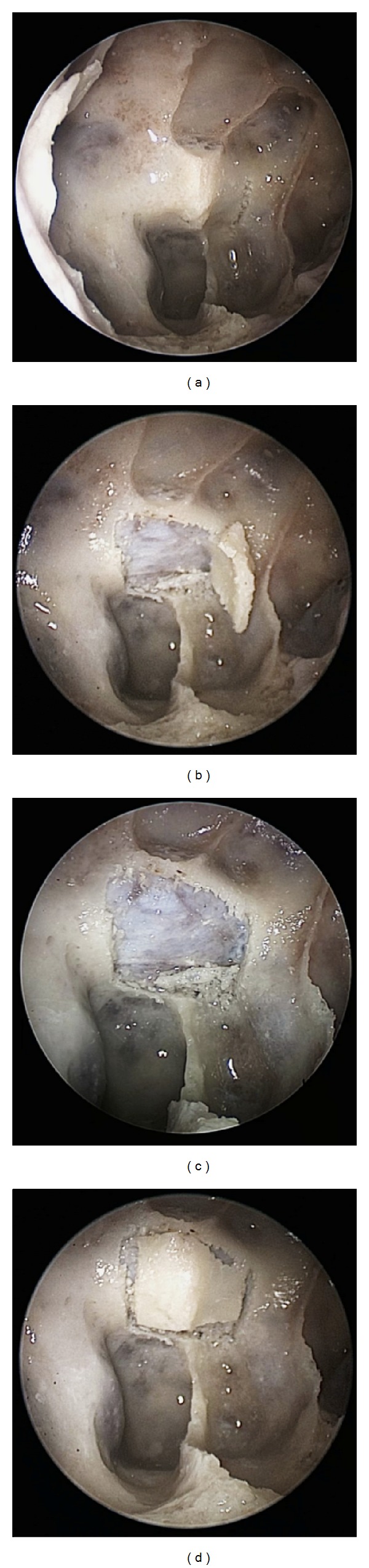
(a) Endoscopic view onto the sellar floor with the primary vertical incision performed on the left. (b) All four incisions are performed with a small bony bridge left for the bone flap to be tilted away like a swinging door. (c) Sellar floor with bone flap removed for demonstration of intact dura. (d) Repositioned bone flap.

**Figure 3:**
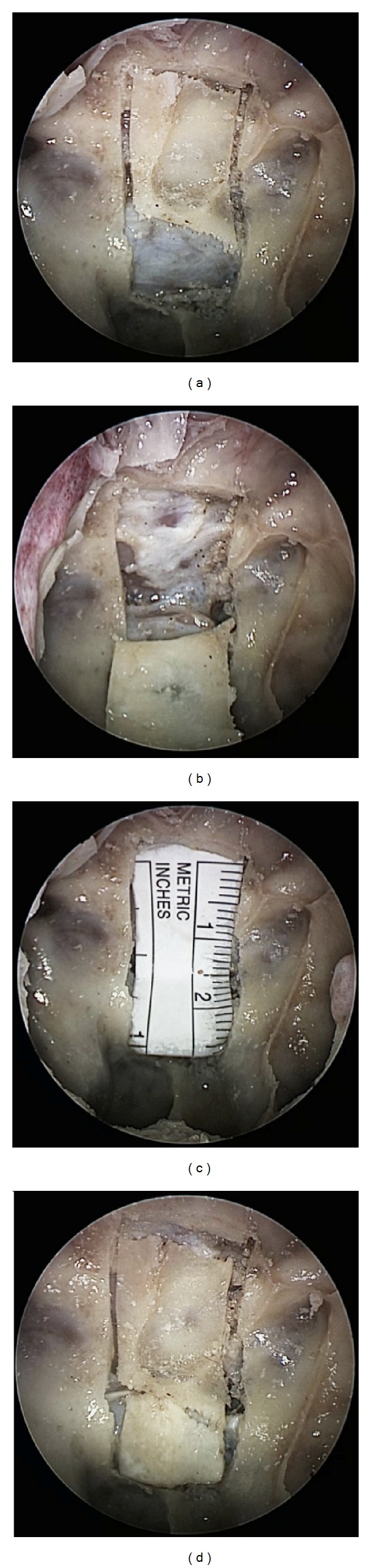
(a) Endoscopic view onto bone flap extended to a transplanum approach with all incisions performed, the sellar bone flap has. (b) Transplanum bone flap tilted inferiorly, a thinned out bone bridge was left at the inferior left corner to act as a door swing. (c) The transplanar bone flap is removed and a measuring scale was put as an “underlay” epidurally to visualize the size of the flap. (d) Both bone flaps are repositioned in the sella and sphenoidal plane, respectively.

On five cadaveric specimens the piezoelectric osteotome (Synthes Gmbh Piezoelectric System, Satelec, Merignac cedex, France, CE 0459) with specially designed slim handpiece and tips for endoscopic approaches was used (Figure 1). The tip is 9 mm long and has a semicircular shape. with small prongs at its top. First, a classical endoscopic approach to the sphenoid sinus through the natural ostia with resection of the anterior sphenoid wall and the posterior superior septum was performed. After visualization and identification of the sellar floor, a mucosal flap was created to expose the bone. The flap was lateralized for later reposition. Then with a frequency of 28 kHz a bone flap in the sellar floor was created using the piezoelectric device under constant irrigation and endoscopic visualization. The mucosa or thinned out bone at one margin of the flap can be preserved to act as a fixation like in a swinging door and to further maintain nutrition of the bone flap (Figures 2(a)–2(d)). After the access to the sella, an extension of the approach to the sellar tuberculum and the sphenoidal plane was performed (Figures 3(a)–3(d)). Finally, a rectangular or hourglass shaped bone flap was created.

The device makes micromovements between 60 and 200 *μ*m/s [10]. The tip was targeted in a 90° angle to the surface of the bone. Gentle longitudinal movements were performed with slight pressure. Duration for creating the bone flap was measured as well as temperature at the areas where the device had contact to the mucosa (thermal probes by (OMEGA HH501AJK, Sensotec, Feldkirchen/Graz, Austria).

The study was performed in accordance with the local institutional review board and the Institute of Anatomy of the Medical University of Graz.

## 3. Results

**Figure 4:**
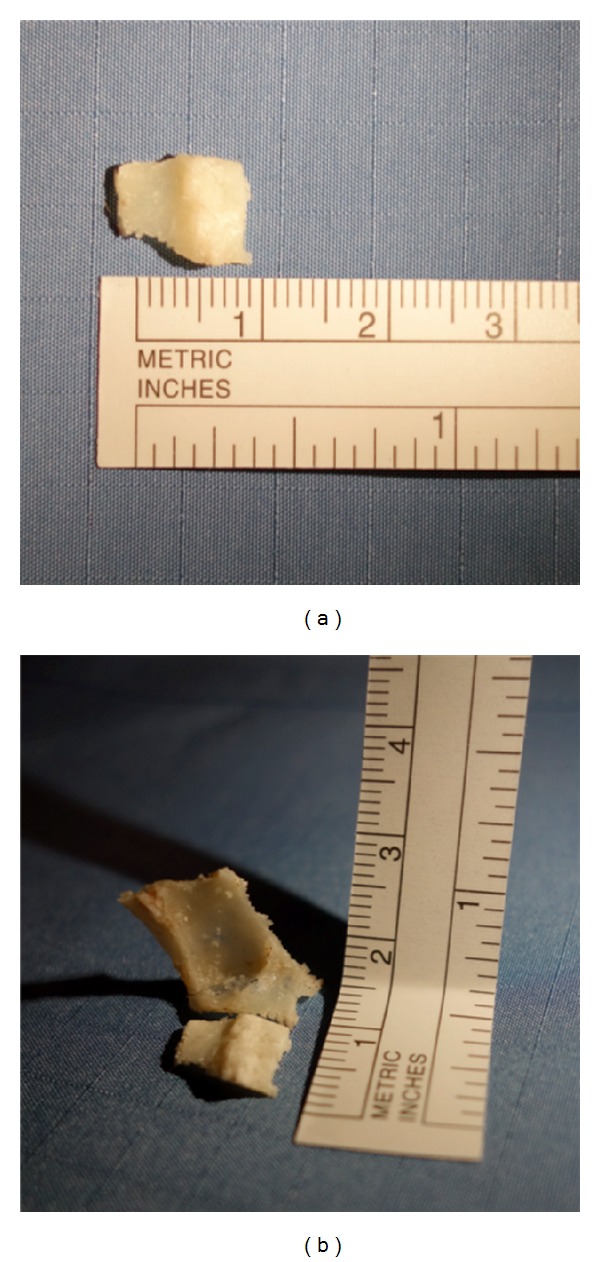
Extracorporal view of the sellar bone flap (a) and both flaps (b) with a measuring scale.

In 5 cadaveric specimens a bone flap could be created with appropriate size (~1 cm^2^) for accessing the intrasellar space. Enlarging the flap for extended transtubercular/transplanum approaches up to a size of 3–5 cm² was possible without breaking the bone, which was repositioned after the intervention and covered by mucosa (Figure 4). In case of sellar access only, a mucosal or bony bridge (with bone thinned out by the piezotome) was preserved serving as a “swinging door.” The dura was not injured by the piezoelectric osteotome. The loss of bone in the incision lines (<1 mm) was minimal. Throughout the procedure excellent endoscopic visualization could be achieved as constant irrigation had a cleansing effect of the surgical field. Both, the suction and the piezoelectric device could be maneuvered by a single surgeon with enough space for movements intranasally while a second surgeon was guiding the endoscope. No limitations regarding maneuverability were experienced and surgical freedom was not impaired as long as the endoscope's lens remained clean. The mucosal temperature at contact areas did not rise above the cadavers' original temperature (26°C). Duration for creating the bone flap in the sellar floor was around 3-4 minutes.

## 4. Discussion

Endoscopic transsphenoidal skull base surgery has become the gold standard for treating pituitary adenomas and various other pathological entities [2]. In most cases ENT surgeons and Neurosurgeons work together in a four-handed technique guiding the endoscope and manipulating instruments at the same time. The nose and paranasal sinuses are used as a corridor to access skull base. Until now exposure of the intracranial lesions requires resection of the bony skull base up to the required extent and incision of the dura. After the resection of the lesion the skull base needs to be reconstructed. In intra- and suprasellar pituitary adenomas where the sellar diaphragm stays intact reconstruction with with hemostyptic material only is sufficient in the majority of cases. In the forthcoming weeks the nasal mucosa will regrow over the defect. On occasions where the sellar diaphragm or the dura at skull base needs to be incised or even resected, reconstruction can be a problem and postoperative CSF leaks may occur. An elegant way to close large skull base defects is the creation of a nasoseptal flap described by Kassam et al. [6] and Fortes et al. [12]. However, the reconstruction as in external craniotomies by means of bone flaps is difficult to accomplish until now.

Apart from malignancies or meningiomas where infiltration of bone is possible and thus its resection is mandatory to obtain safe margins the solution would not be to resect the bone at skull base, but to create flaps as in external craniotomies. The only possibility to create bone flaps as of today would be by means of circular top knives in case of thin bone or hammer and chisel in thicker bone bearing the risk of uncontrolled bone fracturing and accidental injury of vascular and cerebral structures. Contrary to these instruments piezoelectric osteotomes with especially designed tips for endoscopic approaches could be used. This technology allows 28.000 micromovements per second of the piezocrystals that cut bone leaving soft tissue unharmed [9, 10]. The technique was mainly applied in maxillofacial surgery but was recently used for transcranial craniotomies too. Hollstein et al. [8] proved the feasibility of a variety of ultrasonic osteotomes on rabbits' skulls. With all devices thin, straight resection lines could be achieved which is a prerequisite for skull base craniotomies. Gleizal et al. [11] compared injuries of dura with piezosurgery and mechanical saws after craniotomy. Despite the fact that dural injury was reported in the past, the modification of tip design towards rounded edges overcomes this problem, and thus piezosurgery can be considered as safe in experienced hands. Nordera et al. [9] described external orbital decompression with piezosurgery which is advantageous in the vicinity of critical structures like optic nerve and bulb.

Another advantage described was the “cavitation effect” which creates bubbles and saturated steam in the irrigation liquid enhancing the mechanical properties of the device. Furthermore, this has a cleaning effect of the bone margins from blood [9].

A disadvantage of the technology is that the osteotomies take longer as compared to classical saws. This applies to thick bone whereas at skull base the bone is usually thin which would not have a negative effect on operating time [11]. Above this, saws up to now cannot be used endonasally alongside an endoscope. Moreover, the time spent for creation of a nasoseptal flap in large skull base defect reconstruction must be considered. However, after having undergone a learning curve for handling the instrument properly—which at the beginning was time consuming—the time measured in the present study did not relevantly prolong the procedure. Surgical freedom and maneuverability of instruments were not impaired in this series. Possible problems due to individual anatomical variations need to be assessed once this technique is applied clinically on large case series.

To rule out any thermal injury to the nasal mucosa along the corridor caused by the uncovered metal shaft of the piezoelectric instrument, temperature was measured in our study. It did not rise above physiological levels. Any temperature sensation perceived by the surgeon's hand was due to high frequency oscillations provoking a stimulus to temperature receptors of the skin and seized instantly after stopping the system. Measuring the handpiece did not reveal elevated temperatures.

To our knowledge this study is the first to use piezosurgery for endoscopic transnasal craniotomies and proves its feasibility in five cadaveric specimens. We created bone flaps with straight margins which stayed intact and could later be repositioned (Figure 2). The advantage is a reconstruction of the skull base comparable to standard transcranial craniotomies.

Further studies should be designed as an “in vivo” experiment to further evaluate the technique's safety and particularly postoperative healing a well as CSF leak rates. Hence, future trials will be targeted to clinical application of piezoelectric endoscopic transnasal craniotomies (PETCs) and the development of fixation techniques of the bone flap by means of, for example, microscrews, resorbable “rivets,” or the application of the “gasket seal” procedure described by Garcia-Navarro et al. [13].

Once this technique is established for endoscopic transnasal skull base approaches a better stability of reconstruction and potential reduction of postoperative CSF leaks could be provided for the future.

## 5. Conclusion

The piezoelectric endoscopic transsphenoidal craniotomy (PETC) is feasible in a cadaveric model. By this technique a reconstruction of the anterior skull base comparable to transcranial approaches can be achieved. Clinical studies will be performed to prove this hypothesis and future developments should be targeted onto fixation of the bony graft in situ by means of novel fixation devices.
